# Significance of bone marrow fibrosis in acute myeloid leukemia for survival in the real-world

**DOI:** 10.3389/fonc.2022.971082

**Published:** 2022-10-07

**Authors:** Xia Zhang, Fang Wang, Jifeng Yu, Zhongxing Jiang

**Affiliations:** Department of Hematology, First Affiliated Hospital of Zhengzhou University, Zhengzhou, China

**Keywords:** acute myeloid leukemia, bone marrow fibrosis, overall survival, prognosis, complete remission

## Abstract

Acute myeloid leukemia (AML) is a highly heterogeneous hematologic malignancy characterized by the proliferation of myeloid blasts. Bone marrow fibrosis (BMF), characterized by increased deposition of reticulin or collagen fibers, can occur in AML. International authoritative guidelines do not mention AML patients with BMF and the reported studies are inconsistent. Therefore, we retrospectively analyzed the clinical data of newly diagnosed AML patients in our hospital and compared the clinical characteristics, gene mutations and prognosis of AML patients with or without BMF. We found AML patients with BMF tended to be older, were more prone to hepatosplenomegaly, their level of β2-MG was higher and they often had karyotypes associated with a poor prognosis. The proportion of AML patients without BMF was high in the intermediate-risk group and low in the high-risk group. The mutation rates of ASXL1 and TET2 genes were higher and that of CEBPA was lower in the BMF group. Multivariate analysis showed BMF had independent prognostic significance. AML patients without BMF had higher CR/CRi rate, and the time of hematopoietic recovery in patients achieving CR/CRi was longer in BMF group. The degree of BMF, prognostic level and blasts in peripheral blood were independent risk factors for CR/CRi in newly diagnosed AML. AML patients in the BMF group, especially those with BMF ≥ 2, had a lower OS rate. In age<60 years old group, the higher the degree of BMF was, the shorter the median survival time and the lower the OS rate. In age ≥ 60 years old group, the median survival time in the BMF-1 and the BMF-2/3 groups was shorter. For AML with low, intermediate and high risk, there was always a lower OS rate in patients with BMF. The median survival of AML patients decreased with an increasing degree of BMF in different risk stratifications. BMF had no effect on OS of AML patients with HSCT. In conclusion, AML patients with BMF have a poor prognosis, and BMF was an independent prognostic factor for OS. The assessment of BMF was of great significance for the treatment efficacy and prognosis of newly diagnosed AML.

## Introduction

Acute myeloid leukemia (AML) is a highly heterogeneous hematologic malignancy characterized by the proliferation of myeloid blasts or progranulocytes that fail to undergo normal differentiation. The pathogenesis of AML is mainly attributed to chromosomal translocations and mutations of the genes involved in hematopoietic proliferation and differentiation, which results in the accumulation of poorly differentiated myeloid cells (1). The bone marrow microenvironment (BMM) is a complex network composed of blood vessels, nervous systems, hematopoietic cell populations, stromal cell populations, bone marrow adipocytes, cytokines and adhesion molecules and extracellular matrix (ECM) (2). Damage to stromal cell populations and the ECM may lead to bone marrow fibrosis (3). However, many recent studies found that genetic lesions and BMMs that could not regulate hematopoietic stem cells (HSCs) were responsible for the transition to leukemia stem cells (LSCs) (4). In turn, the transformed LSCs promoted the remodeling of the BMM (5). Consequently, the BMM is considered to play a crucial role in both hematopoiesis and leukemogenesis.

Bone marrow fibrosis (BMF) is characterized by increased deposition of reticulin fibers or collagen fibers (6). However, BMF, observed in any type of AML, is more frequent in acute megakaryocytic leukemia (AML-M7) (7, 8). In recent years, there have also been some reports about chronic myeloid leukemia (CML) (9) and myelodysplastic syndrome (MDS) (10) combined with BMF. Tumor aggression and poor prognosis were found to be correlated with the degree of tissue fibrosis and level of stromal stiffness in solid tumors (11). However, the study of BMF in hematological malignancies is relatively rare. Research reports marrow fibrosis is a factor predictive of a poor prognosis in patients with MDS (12). International authoritative guidelines, such as the NCCN clinical practice guidelines (13), ESMO clinical practice guidelines (14), World Health Organization (WHO) (15) and ELN (16) guidelines, do not mention the gene mutation and prognosis analysis of AML patients with BMF. Studies have reported on this issue, but the results are inconsistent. One study by Manoharan A et al. showed that BMF did not affect the overall survival (OS) of patients with AML and that effective anti-leukemia treatment could reverse BMF (17). However, another study found a poor prognosis in AML patients with BMF (18). Therefore, we retrospectively analyzed the clinical data of newly diagnosed AML patients in our hospital and compared the clinical characteristics, gene mutations and prognosis of newly diagnosed AML patients with or without BMF. In order to clarify the influence of BMF on the efficacy and prognosis of newly diagnosed AML, further explore whether AML with BMF can be regarded as an independent clinicopathological feature or be included in prognosis stratification and guide such patients to make more reasonable treatment plans.

## Materials and methods

### Patients and clinical procedures

This retrospective study was approved by the Ethics Committee of the First Affiliated Hospital of Zhengzhou University, and written informed consent was obtained from all subjects or their guardians. Clinical samples of hospitalized patients were collected from December 2014 to September 2021. A total of 605 newly diagnosed AML patients were enrolled in our study. All patients underwent examinations of morphology, immunology, cytogenetics, molecular biology and bone marrow biopsy. The diagnosis and prognosis of AML were made according to the guidelines and the WHO classification systems (15). A bone marrow biopsy was performed in 190 AML (non-acute promyelocytic leukemia, non-APL) patients. The CAG regimen (low-dose cytarabine 10 mg/m^2^ every 12 hours on days 1-14, aclarubicin (14 mg/m^2^ every day on days 1-4 and granulocyte colony-stimulating factor 200 μg/m^2^ every day on days 1-14) as induction therapy for the treatment of poor-prognosis AML (19). Other AML patients received conventional “7 + 3” regimens: DA (cytarabine 200 mg/m^2^ every day on days 1-7, daunorubicin 60 mg/m^2^ every day on days 1-3), IA (cytarabine 200 mg/m^2^ every day on days 1-7, idarubicin 12 mg/m^2^ every day on days 1-3), MA (cytarabine 200 mg/m^2^ every day on days 1-7, mitoxantrone 12 mg/m^2^ every day on days 1-3), which was used for induction chemotherapy (13). Consolidation chemotherapy was conducted after complete remission (CR), which included the original induction chemotherapy plus intermediate- or high-dose cytarabine. According to the NCCN guidelines, lumbar puncture (LP) and intrathecal injection were performed to prevent or treat central nervous system involvement in AML patients. LP is not recommended in asymptomatic patients at diagnosis. Patients with headache, confusion, and paresthesia should be examined first by radiology (CT/MRI) to rule out neurological bleeding or mass. If there is no evidence of intracranial hemorrhage, LP can be performed after correcting the coagulation disorder and platelet transfusion. If leukemic cells are found in cerebrospinal fluid, LP and intrathecal injection should be performed with systemic chemotherapy. If symptoms persist but cerebrospinal fluid is normal, LP should be performed (13). The hematopoietic stem cell transplantation (HSCT) was performed in accordance with clinical guidelines based on the classification and AML risk stratification (13). Complete remission and progression of the disease were defined according to references (20–22).

### Bone marrow biopsy, pathological film and reticular fiber staining

Qualified bone marrow tissue (1-2 cm in length and more than 0.2 cm in diameter) was removed. Then the tissues were fixed in 4% paraformaldehyde. After decalcification, dehydration and paraffin embedding, the wax blocks were thinly sliced. The paraffin sections were stained with hematoxylin-eosin and immunohistochemistry. The reticular fibers were stained by the Gomori method. The radiographs were reviewed by professional physicians of the Institute of Hematology in our hospital. The grading criteria of bone marrow fibrosis were in accordance with the 2005 European consensus on grading bone marrow fibrosis (23).

### Other indicators of detection

Bone marrow aspiration was subjected to chromosome karyotype analysis, second-generation sequencing, preliminary screening and prognostic gene detection to assess the AML patient’s prognosis and guide the treatment. General information about the patients (gender, age) was also recorded. Routine blood tests, the percentage of peripheral blood/bone marrow primitive cells, lactate dehydrogenase (LDH), β2 microglobulin (β2-MG), blood type and hepatosplenomegaly were also assessed.

### Follow up

All cases were followed up to May 30, 2022. Follow-up data were obtained from inpatient and outpatient medical records. Patients who died during the follow-up period were confirmed according to the course of the disease records or by telephone contact with the patient’s family members. Survival time was calculated from diagnosis to death or from diagnosis to May 30, 2022.

### Statistical analysis

Data were analyzed using SPSS 26.0 software (SPSS, Chicago, IL, US). Quantitative data were compared using a t-test (for a normal distribution) or a nonparametric test (Mann–Whitney Test, not a normal distribution). The chi-squared (χ2) test was used for comparison of the categorical data. First, a t-test was used for univariate analysis. Second, a nonconditional logistics regression model was used for multivariate analysis. The Kaplan–Meier method was used to plot cumulative survival curves. A Cox regression model was used for multivariate analysis of overall survival. Only the independent variables that P <0.05 in the univariate analysis in the previous step, were subjected to multivariate regression analysis. Data analysis was performed using GraphPad Prism 9 software (GraphPad Software, La Jolla, California). P<0.05 was considered statistically significant.

## Results

### Patient characteristics

In this retrospective study, there were a total of 190 newly diagnosed patients with AML (non-APL). Among them, there were 130 AML patients with BMF and 60 without BMF. There were more men than women in both groups (AML with BMF vs. without BMF, 72/58 vs. 31/29). The median age was 51.9 and 42.5 years old among AML patients with and without BMF, and there was a significant difference between the two groups (P = 0.000). The level of serum β2-MG in AML patients with BMF was higher than that in those without BMF, and the difference was statistically significant (P = 0.000). Hepatosplenomegaly was more common in AML patients with BMF than in those without BMF (39.50% vs. 22.00%) (P = 0.045). In the high-risk cytogenetics group, AML patients with BMF accounted for a higher proportion (13.59% vs. 2.22%), and the difference was statistically significant (P = 0.006). Prognostic risk stratification was significantly different in AML patients with or without BMF (P =0.036). In the intermediate group, the proportion of AML without BMF was higher than AML with BMF (58.33% vs. 21.54%), and the P value was 0.000. Conversely, the proportion of AML without BMF was lower than AML with BMF in the poor prognosis group (18.33% vs. 61.54%), and the P value was 0.000. However, there was no significant difference in the number of AML patients with or without BMF in the good prognosis group. At the same time, there was no significant difference between blasts in the peripheral blood and bone marrow between the two groups. There was no significant difference in white blood cell count, hemoglobin and platelet count in the peripheral blood, LDH, AST, ALT, α1-MG, D dimer, ferritin and blood type between the two groups (P > 0.05). There was no therapy related AML in either group, but there was secondary AML in both groups (AML with BMF vs. without BMF, 13/130 vs. 5/60), and there was no significant difference between the two groups (P = 0.796).

Details of the clinical features are listed in **Table 1**.

**Table 1:** Comparison of clinical features in newly diagnosed AML patients with and without BMF.

Clinical characteristics	AML with BMF (130)	AML without BMF(60)	P value
Gender (male/female)	72/58	31/29	0.639
Age, year, median (range)	51.9 (15∼82)	42.5 (14∼73)	0.000
WBC (×10^9^/L), median (range)	35.1 (0.5∼332)	22.2 (0.85∼153.6)	0.051
HB (g/L), median (range)	74.9 (11∼142)	80.6 (42∼152)	0.099
PLT (×10^9^/L), median (range)	95.3 (2∼1262)	76.6 (2∼1510)	0.513
Blasts in PB (%), median (range)	37.3 (0∼98)	40.5 (0∼96)	0.514
Blasts in BM (%), median (range)	51.7 (20∼95.6)	57.3 (20∼93.2)	0.101
LDH(IU/L), median (range)	611.0 (109∼2518)	478.6 (110∼2111)	0.131
ALT(U/L), median (range)	26.7 (5∼140)	26.0 (4∼119)	0.875
AST(U/L), median (range)	26.9 (5∼161)	21.5 (8∼89)	0.098
β2-MG (mg/L), median (range)	2.5 (0.61∼8.2)	1.58 (0.62∼3.33)	0.000
α1-MG (mg/L), median (range)	21.1 (7.81∼49)	20.3 (10∼29)	0.605
D dimer (mg/L), median (range)	5.9 (0.06∼430.6)	1.59 (0.09∼12.83)	0.523
Ferritin (ng/mL), median (range)	1375.1 (37∼10738)	826.3 (10.8∼2384.1)	0.215
Blood type (%)			0.238
A+	19.83	32.14	0.073
B+	34.71	32.14	0.737
AB+	11.57	14.29	0.611
O+	33.88	21.43	0.092
Hepatomegaly/Splenomegaly (%)	39.50	22.00	0.045
Cytogenetics (%)			0.140
Low risk	9.71	22.22	0.597
Intermediate risk	76.70	75.56	0.881
High risk	13.59	2.22	0.006
Prognostic level, n (%)			0.036
Low risk	22 (16.92%)	14 (23.33%)	0.320
Intermediate risk	28 (21.54%)	35 (58.33%)	0.000
High risk	80 (61.54%)	11 (18.33%)	0.000
AML diagnosis			
*De novo*	117 (90.00%)	55 (91.67%)	0.796
Secondary AML	13 (10.00%)	5 (8.33%)	0.796

WBC, white blood cells; HB, hemoglobin; PLT, platelet; PB, peripheral blood; BM, bone marrow; BMF, bone marrow fibrosis; AML, acute myeloid leukemia; BMF, bone marrow fibrosis.

### Gene mutation analysis in newly diagnosed AML patients with and without BMF

The results of gene mutation analysis in the two groups are shown in **Table 2**. The mutation rates of the ASXL1 and TET2 genes were higher in the AML with BMF group than in the AML without BMF group (P = 0.004 and 0.048, respectively). However, the mutation frequency of CEBPA was significantly lower than that of patients without BMF (P = 0.000). Other mutated genes, such as FLT3-ITD, FLT3-TKD, TP53, DNMT3A, and NPM1, showed no significant difference between the two groups (P > 0.05).

**Table 2:** Gene mutation analysis in newly diagnosed AML patients with and without BMF.

Gene name	AML with BMF, n (%)	AML without MF, n (%)	P value
FLT3-ITD	21 (16.15%)	8 (13.33%)	0.618
FLT3-TKD	5 (3.85%)	7 (11.67%)	0.054
CEBPA	10 (7.69%)	21 (35%)	0.000
NPM1	20 (15.38%)	6 (10%)	0.287
C-kit	8 (6.15%)	5 (8.33%)	0.583
TP53	5 (3.85%)	2 (3.33%)	0.862
RUNX1	10 (7.69%)	2 (3.33%)	0.190
ASXL1	40 (30.77%)	8 (13.33%)	0.004
DNMT3A	18 (13.85%)	5 (8.33%)	0.244
IDH1	1 (0.77%)	3 (5%)	0.155
IDH2	6 (4.62%)	2 (3.33%)	0.684
SF3B1	3 (2.31%)	2 (3.33%)	0.683
U2AF1	9 (6.92%)	3 (5%)	0.615
SRSF2	7 (5.38%)	3 (5%)	0.913
ZRSR2	1 (0.77%)	2 (3.33%)	0.301
EZH2	2 (1.54%)	0 (0)	0.337
TET2	83 (63.85%)	29 (48.33%)	0.048
CBL	8 (6.15%)	2 (3.33%)	0.421
JAK2/V617F	8 (6.15%)	1 (1.67%)	0.097
NRAS	30 (23.08%)	9 (15.00%)	0.177
KRAS	1 (0.77%)	0 (0)	0.498
ETV6	4 (3.08%)	4 (6.67%)	0.320
SETBP1	6 (4.62%)	1 (1.67%)	0.318
GATA2	1 (0.77%)	1 (1.67%)	0.576
IKZF1	0 (0)	1 (1.67%)	0.321

AML, acute myeloid leukemia; BMF, bone marrow fibrosis.

### The impact of different induction therapies on CR/CRi rate and overall survival

In our retrospective study, the standard DA, IA, MA or CAG formula was used for induction chemotherapy in our retrospective study. There were only two AML patients with BMF receiving MA formula, but none in AML without BMF. Consequently, we analyzed the impact of the remaining three induction therapies on complete response (CR)/morphologic complete remission with an incomplete blood count recovery (CRi) rate and overall survival (OS). The CR/CRi rate of the IA, DA and CAG groups in AML with the BMF group was 57.78%, 53.85% and 41.67% (P = 0.446), respectively, and there were no statistically significant differences between any two groups of the three induction therapies (P > 0.05). In AML without the BMF group, the CR/CRi rate in the IA, DA and CAG groups was 85.71%, 62.50% and 75.00% (P = 0.359), respectively. Similarly, there was no statistical difference between any two groups of the three induction therapies in this group (P > 0.05) (**Table 3**).

**Table 3:** Impact of different induction therapies on CR/CRi rate.

Group	IA	DA	CAG	P value
AML with BMF, n (%)	26 (57.78%)	14 (53.85%)	10 (41.67%)	0.446
AML without BMF, n (%)	12 (85.71%)	10 (62.50%)	21 (75.00%)	0.359

AML, acute myeloid leukemia; BMF, bone marrow fibrosis; CR, complete remission; CRi, morphologic complete remission with incomplete blood count recovery.

**Figure 1:**
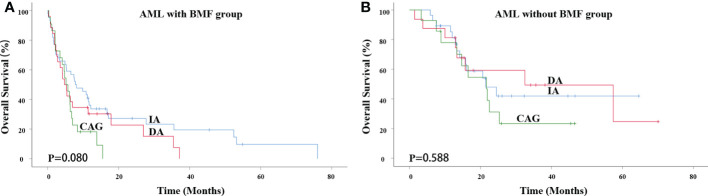
Impact of different induction therapies on OS in primary AML patients. **(A)** Kaplan-Meier curves comparing the OS of patients with BMF receiving induction therapies, such as IA (blue), DA (red) and CAG (green) (7.800 months vs. 4.733 months vs. 5.167 months, P = 0.080). **(B)** Kaplan-Meier curves comparing the OS of patients with BMF receiving induction therapies, such as IA (blue), DA (red) and CAG (green) (21.500 months vs. 32.533 months vs. 21.667 months, P = 0.588). AML, acute myeloid leukemia; BMF, bone marrow fibrosis; OS, overall survival.

The median survival time of the IA, DA and CAG groups in AML with BMF receiving induction therapies was 7.800 months, 4.733 months and 5.167 months, respectively. There was no significant difference among the three groups (χ^2^ = 5.061, P = 0.080) (**Figure 1A**). In AML without BMF group, the median survival time of IA, DA and CAG groups was 21.500 months, 32.533 months and 21.667 months. There was no significant difference among the three groups (χ^2^ = 1.060, P = 0.588) (**Figure 1B**).

### Effect of BMF on the induction remission rate in primary diagnosed AML patients

The CR/CRi rate was 54.25% in AML with BMF and 77.19% in AML without BMF, and there was a significant difference between the two groups (P = 0.004). AML patients without BMF had higher CR rate (AML with the BMF vs. without the BMF, 39.36% vs. 61.40%, P = 0.008). The proportion of induction failure in AML with the BMF group was higher than that without the BMF group (23.40% vs. 7.02%) (P = 0.010). The recovery time of bone marrow hematopoietic function in patients achieving CR/CRi was longer in the BMF group (P = 0.034) (**Table 4**). Multivariate analysis using a non-conditional logistic regression model showed that BMF had independent prognostic significance (P = 0.001). The degree of fibrosis was an independent risk factor for CR/CRi in newly diagnosed AML patients [BMF-2/3 vs. BMF-0, HR, 95% CI, 0.351 (0.194-0.634), P = 0.001; BMF-2/3 vs. BMF-1, HR, 95% CI, 0.189 (0.068-0.521), P = 0.001]. The prognostic level was an independent risk factor for CR/CRi in newly diagnosed AML patients [high risk vs. low risk and intermediate, 0.369 (0.163-0.834), P = 0.017]. Blast in peripheral blood (PB) was a risk factor [blast vs. without blast in PB, 0.098 (0.012-0.800), P = 0.030] (**Table 5**).

**Table 4:** Induced chemotherapy response and recovery time of BMHF in newly diagnosed AML patients with and without BMF.

Treatment response	AML with BMF	AML without BMF	P value
CR/CRi, n (%)	51 (54.25%)	44 (77.19%)	0.004
CR, n (%)	37 (39.36%)	35 (61.40%)	0.008
CRi, n (%)	14 (14.89%)	9 (15.79%)	0.883
PR, n (%)	21 (22.34%)	9 (15.78%)	0.331
Induction failure, n (%)	22 (23.40%)	4 (7.02%)	0.010
Abandoning therapy, (n)	36	3	
Recovery time of BMHF			
CR/CRi ^a^, day, median (range)	26 (13~73)	22 (7~61)	0.034
CR ^b^, day, median (range)	24 (13~73)	22 (7~61)	0.067

AML, acute myeloid leukemia; BMF, bone marrow fibrosis; CR, complete remission; CRi, morphologic complete remission with incomplete blood count recovery; PR, partial remission. ^a^the recovery time of bone marrow hematopoietic function in patients achieving CR/CRi; ^b^the recovery time of bone marrow hematopoietic function in patients achieving CR.

**Table 5:** Univariate and multivariate analyses of CR/CRi for patients with newly diagnosed AML.

Covariates	Univariate		Multivariate	
	HR (95% CI)	P	HR (95% CI)	P
Age^a^	0.433 (0.179-1.047)	0.063		
Gender (male vs. female)	1.266 (0.653-2.457)	0.485		
WBC^b^	0.863 (0.445-1.671)	0.662		
Blasts in PB^c^	0.118 (0.015-0.927)	0.042	0.098 (0.012-0.800)	0.030
Blasts in BM^d^	0.552 (0.056 -5.434)	0.610		
Cytogenetics^e^	0.306 (0.084-1.120)	0.074		
Prognostic level^f^	0.334 (0.169-0.663)	0.002	2.710 (1.199-6.123)	0.017
Bone marrow fibrosis		0.008		0.001
BMF-0 VS BMF-1	0.802 (0.336-1.916)	0.619	0.906 (0.323-2.543)	0.851
BMF-0 VS BMF-2/3	0.344 (0.213-0.555)	0.000	0.351 (0.194-0.634)	0.001
BMF-1 VS BMF-2/3	0.147 (0.057-0.383)	0.000	0.189 (0.068-0.521)	0.001

HR, hazard ratio; CI, confidence interval; ^a^Age < 60 years old versus ≥ 60 years old; ^b^WBC ≤10×10^9^/L versus WBC >10×10^9^/L; ^c^Blasts in peripheral blood versus without blasts; ^d^Blasts in bone marrow >20% versus = 20%; ^e^risk versus intermediate and high risk; ^f^Low and intermediate risk versus high risk. CR, complete remission; CRi, Morphologic complete remission with incomplete blood count recovery; AML, acute myeloid leukemia.

### Overall survival

**Figure 2:**
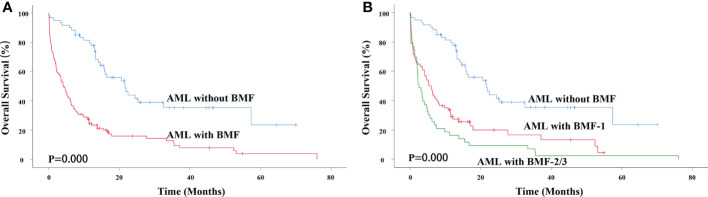
Impact of BMF on OS in primary AML patients. **(A)** Kaplan-Meier curves comparing the OS of patients without (blue) or with BMF (red) (21.667 months vs. 4.200 months, P = 0.000). **(B)** Kaplan-Meier curves comparing the OS of patients without (blue), with BMF-1 (red) or BMF-2/3 (green) (21.667 months vs. 5.400 months vs. 2.533 months, P = 0.000). AML, acute myeloid leukemia; BMF, bone marrow fibrosis; OS, overall survival.

The median survival time of AML without BMF was 21.667 months and that of AML with BMF was 4.200 months. The 3-year overall survival (OS) rate of AML patients without BMF was 35.4% and that of AML patients with BMF was 9.6%. There was a significant difference between the two groups (χ^2 =^ 35.200, P=0.000) (**Figure 2A**). AML with BMF was divided into two subgroups according to the degree of fibrosis: the BMF-1 group and the BMF-2/3 group. The OS of the BMF-1 group and the BMF-2/3 group was compared with that of the AML without BMF group, and there were statistically significant differences among the three groups (χ^2 =^ 41.140, P=0.000). The median survival of the AML without BMF group was 21.667 months, 5.400 months in the BMF-1 group, and 2.533 months in the BMF-2/3 group. The 3-year OS rate of AML patients without BMF was 35.4%, AML patients with BMF-1 was 16.6%, and BMF-2/3 was 2.3% (**Figure 2B**).

Cox multivariate analysis showed that BMF had independent prognostic significance for the OS of primary AML patients (P = 0.000), especially AML patients in the BMF-2/3 group, who had worse OS [HR, 95% CI, 2.203 (1.661-2.924), P = 0.000]. Meanwhile, Cox multivariate analysis showed that age had independent prognostic significance for the OS of primary AML patients (P=0.000), especially age ≥60 years had a worse OS [HR, 95% CI, 2.495 (1.708-3.644), P = 0.000] (**Table 6**).

**Table 6:** Cox regression analysis for overall survival in newly diagnosed primary AML.

Covariates	Univariate		Multivariate	
	HR (95% CI)	P	HR (95% CI)	P
Age^a^	2.628 (1.824-3.786)	0.000	2.495 (1.708-3.644)	0.000
Gender (male vs. female)	1.050 (0.756-1.460)	0.770		
WBC^b^	0.980 (0.704-1.365)	0.906		
Blasts in PB^c^	1.291 (0.655-2.542)	0.461		
Blasts in BM^d^	0.812 (0.330-2.001)	0.651		
Cytogenetics^e^	1.508 (0.827-2.753)	0.180		
Prognostic level^f^	1.761 (1.261-2.458)	0.001	1.004 (0.694-1.451)	0.984
Bone marrow fibrosis		0.000		0.000
BMF-0 VS BMF-1	2.853 (1.850-4.400)	0.000	2.556 (1.602-4.078)	0.000
BMF-0 VS BMF-2/3	2.053 (1.626-2.591)	0.000	2.203 (1.661-2.924)	0.000
BMF-1 VS BMF-2/3	1.531 (1.029-2.278)	0.036	1.577 (1.051-2.366)	0.028

HR, hazard ratio; CI, confidence interval; ^a^Age < 60 years old versus ≥ 60 years old; ^b^WBC ≤10×10^9^/L versus WBC >10×10^9^/L; ^c^Blasts in peripheral blood versus without blasts; ^d^Blasts in bone marrow >20% versus = 20%; ^e^Low risk versus intermediate and high risk; ^f^Low and intermediate risk versus high risk; AML, acute myeloid leukemia.

### Survival by age

**Figure 3:**
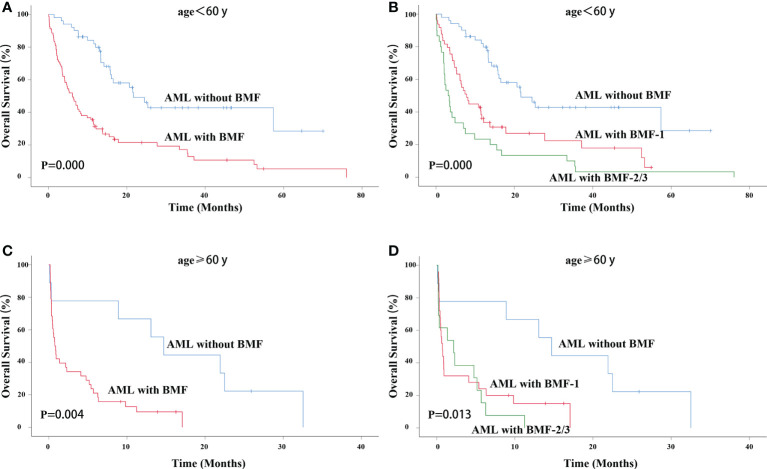
OS in different age groups. OS of primary AML patients (age < 60 years old) with or without BMF **(A)** or BMF subgroups **(B)**. OS of primary AML patients (age ≥ 60 years old) with or without BMF **(C)** or BMF subgroups **(D)**. AML, acute myeloid leukemia; BMF, bone marrow fibrosis; OS, overall survival.

For AML patients younger than 60 years old, the AML with BMF group had a lower OS rate (3-year OS rate: 42.8% vs. 12.9%, χ^2^ = 24.276, P = 0.000). The median survival time of AML without BMF was 21.667 months while that of AML with BMF was 6.133 months (**Figure 3A**). The OS of the BMF-1 group and the BMF-2/3 group was compared with that of the AML without BMF group for AML patients < 60 years old, and there were statistically significant differences among the three groups (χ^2^ = 31.205, P = 0.000). The median survival of the AML without BMF group was 21.667 months, 7.800 months in the BMF-1 group, and 3.133 months in the BMF-2/3 group. The 3-year OS rate of AML patients without BMF was 42.8% and that of AML patients with BMF-1 was 22.4%, with BMF-2/3 being 3.3% (**Figure 3B**).

For AML patients older than 60 years old, the AML with BMF group also had a lower OS rate (3-year OS rate: 0 vs. 0, χ^2^ = 8.215, P = 0.004). The median survival time of AML without BMF was 14.700 months and that of AML with BMF was 0.767 months (**Figure 3C**). The OS of the BMF-1 group and the **B**MF-2/3 group was compared with that of the AML without BMF group for AML patients ≥ 60 years old, and there were statistically significant differences among the three groups (χ^2^ = 8.697, P = 0.013). The median survival of the AML without BMF group was 14.700 months, 0.733 months in the BMF-1 group, and 2.167 months in the BMF-2/3 group. All of the 3-year OS rate in BMF subgroups was 0 (**Figure 3D**).

### Survival by different prognostic levels

**Figure 4:**
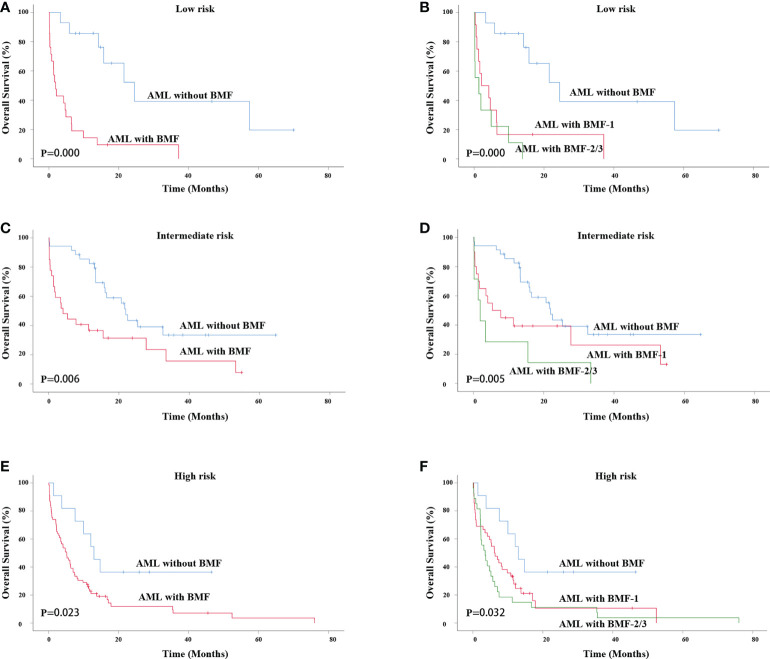
Survival analysis of patients with different risk stratifications. OS of primary AML patients (low risk) with or without BMF **(A)** or BMF subgroups **(B)**. OS of primary AML patients (intermediate risk) with or without BMF **(C)** or BMF subgroups **(D)**. OS of primary AML patients (intermediate risk) with or without BMF **(E)** or BMF subgroups **(F)**. AML, acute myeloid leukemia; BMF, bone marrow fibrosis; OS, overall survival.

Next, we performed survival analysis for AML patients with or without BMF at different risk stratifications. For primary AML patients with low risk, the AML with BMF group also had a lower OS rate (3-year OS rate: 39.2% vs. 9.5%, χ^2^ = 16.533, P = 0.000). The median survival time of AML without BMF was 24.500 months while that of AML with BMF was 1.933 months (**Figure 4A**). The OS of the BMF-1 group and the BMF-2/3 group was compared with that of the AML without BMF group for AML patients, and the results showed statistically significant differences among the three groups (χ^2^ = 19.709, P = 0.000). The median survival of the AML without BMF group was 24.500 months, 2.133 months in the BMF-1 group, and 1.367 months in the BMF-2/3 group. The 3-year OS rate of AML patients without BMF was 39.2% and that of AML patients with BMF-1 was 16.7%, with BMF-2/3 being 0 (**Figure 4B**).

For AML patients with an intermediate risk, the AML with BMF group also had a lower OS rate (3-year OS rate: 33.5% vs. 15.7%, χ^2^ = 7.571, P = 0.006). The median survival time of AML without BMF was 21.933 months and that of AML with BMF was 4.100 months (**Figure 4C**). The OS of the BMF-1 group and the BMF-2/3 group was compared with that of the AML without BMF group, and the results showed statistically significant differences among the three groups (χ^2^ = 10.452, P = 0.005). The median survival of the AML without BMF group was 21.933 months, 5.400 months in the BMF-1 group, and 1.933 months in the BMF-2/3 group. The 3-year OS rate of AML patients without BMF was 33.5% while that of AML patients with BMF-1 was 26.3%, with BMF-2/3 being 0 (**Figure 4D**).

For AML patients with high risk, the AML with MF group also had a lower OS rate (3-year OS rate: 36.4% vs. 7.2%, χ^2^ = 5.161, P = 0.023). The median survival time of AML without BMF was 12.967 months while that of AML with BMF was 5.167 months (**Figure 4E**). The OS of the BMF-1 group and the BMF-2/3 group was compared with that of the AML without BMF group, there were statistically significant differences among the three groups (χ^2^ = 6.910, P = 0.032). The median survival of the AML without BMF group was 12.967 months, 6.133 months in the BMF-1 group, and 3.500 months in the BMF-2/3 group. The 3-year OS rate of AML patients without BMF was 36.4%, while that of the AML patients with BMF-1 was 10.6%, with BMF-2/3 being 3.7% (**Figure 4F**).

### Survival by HSCT

**Figure 5:**
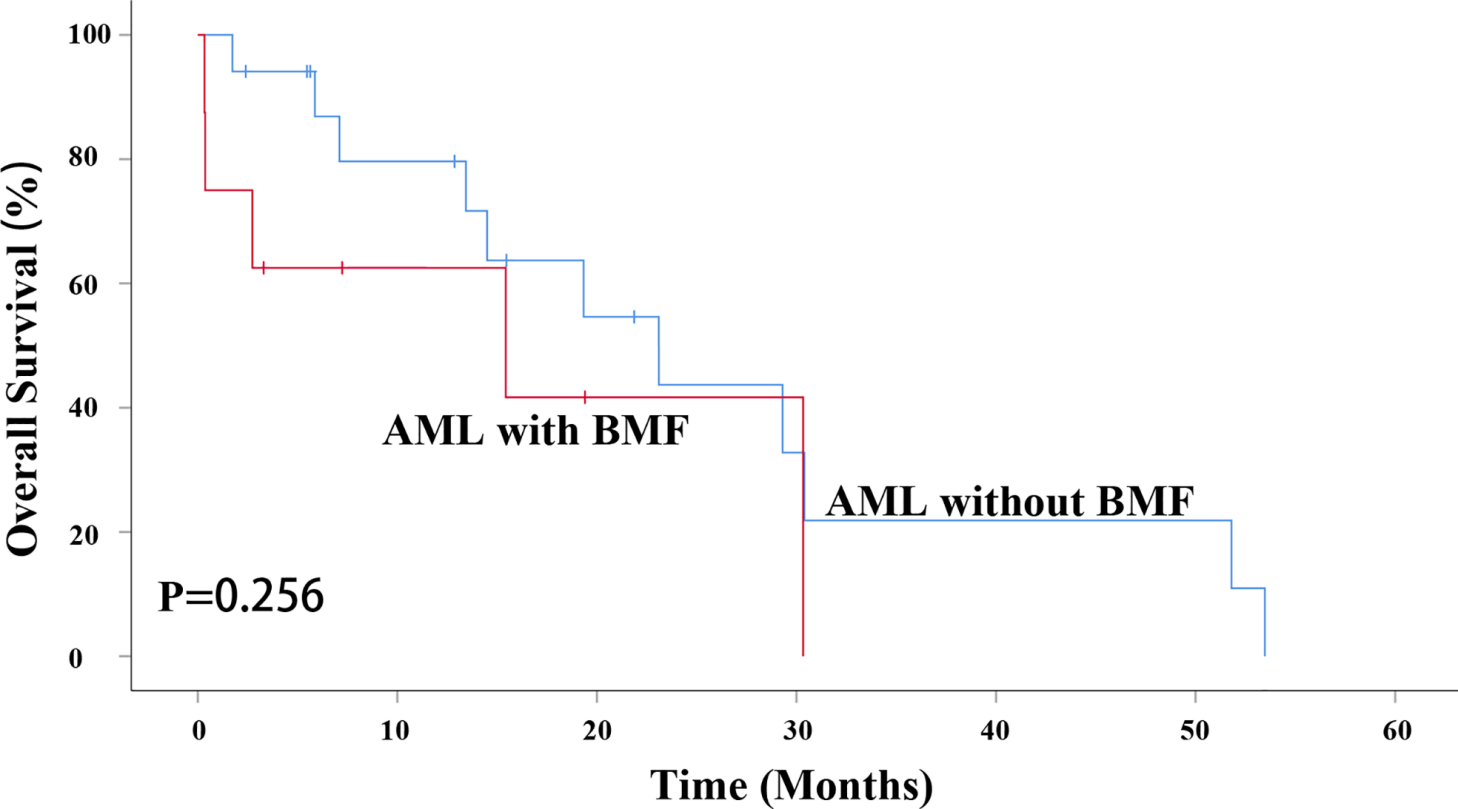
BMF had no effect on OS in AML patients undergoing HSCT (P = 0.256). AML, acute myeloid leukemia; BMF, bone marrow fibrosis; OS, overall survival; HSCT, hematopoietic stem cell transplantation.

However, due to the influence of patient status, family economic status, donor availability and other factors, not all of the AML patients with intermediate and high risk received allogeneic transplant. A total of 8 AML patients with BMF and 17 without BMF underwent transplantation. BMF had no effect on OS in AML patients undergoing HSCT (P = 0.256). The median survival time of AML without BMF was 23.100 months while that of AML with BMF was15.433 months. The 3-year OS rate of AML patients without BMF was 36.4%, while that of AML patients with BMF being 0 (**Figure 5**).

## Discussion

The most frequent type of leukemia associated with the syndrome of bone marrow fibrosis is acute megakaryoblastic leukemia (AMKL) (8, 24, 25), but as has been shown in this study, it is also present in other types of AML. Islam et al. reviewed the clinical features of 34 patients with AML, approximately one-third (12/34) of whom had various degrees of BMF at the time of their diagnosis with AML. In addition, a previous study showed that fibrosis did not affect the regeneration of the hematopoietic system (26). However, another study showed that engraftment was significantly delayed in MDS patients with fibrosis. Overall, bone marrow fibrosis had no significant effect on the OS of MDS patients with HSCT with a low International Prognostic Scoring System (IPSS) score, but the OS, relapse-free survival (RFS), and non-relapse mortality (NRM) between MDS patients (int-2 or high-risk disease) with and without fibrosis were inferior (12).

The pathogenesis of AML with BMF remains unclear. It has been suggested that the abnormal proliferation of BMF is a secondary reaction to the clonal proliferation of hematopoietic cells (27). Bone marrow stromal cells consist of endothelial cells, adipocytes, macrophages and reticular cells. The deposition of reticulin and collagen fibrosis in the bone marrow of patients with BMF is mediated by bone marrow fibrosis hematopoietic stem/progenitor cells, resulting in an impaired hematopoietic microenvironment that is conducive to malignant and abnormal hematopoiesis (6). Dilly et al. found that stromal cells such as reticular cells and vascular endothelial cells were increased in both acute and chronic granulocyte tumors, and most granulocyte tumors increased the synergistic stimulation of stromal cells and tumor cells (28). Although the exact mechanism of myelofibrotic progression in AML is unclear, one study suggested that certain factors are released by proliferating megakaryocytes because they are unable to store these factors (platelet-derived growth factor, fibroblast growth factor, platelet factor-4, transforming growth factor-β and beta-thromboglobulin) in defective α particles, which promote the growth of bone marrow fibroblasts (29). Other studies have also confirmed platelet-derived growth factor modified by malignant megakaryocytes and its leakage into the BMM promotes fibroblast activity (30, 31). Collagenase inhibitor, platelet factor 4 (32) and transforming growth factor, which promote collagen synthesis (33), play an important role in the progression of BMF (34). Leukemia cells express specific growth factor proteins, platelet-derived growth factor, transforming growth factor and fibronectin in extramedullary tumors and may selectively regulate tumor formation (34).

There are many studies on AMKL (34–36) but few studies on AML patients with or without BMF. In terms of clinical characteristics, AML patients with BMF tended to be older. AML patients with BMF were more prone to hepatosplenomegaly, which is consistent with previous reports (34, 36). β2-MG was higher in AML patients with BMF than in those without BMF. Newly diagnosed AML patients with BMF often have poor prognosis karyotypes. We compared the two groups of patients according to their different prognostic subgroups. The proportion of AML patients without BMF was high in the intermediate-risk group and low in the high-risk group. There was no significant difference in white blood cell count, hemoglobin and platelet count, LDH, AST, ALT, α1-MG, D dimer, ferritin, blood type and AML diagnosis.

AML patients with epigenetic modification gene ASXL1 mutations, considered an independent predictor of a poor outcome, affect 5-11% of AML patients and are especially common in older, male and secondary AML patients (37–39). A TET2 mutation, an unfavorable prognostic factor in AML patients with intermediate-risk cytogenetics, especially when it is combined with other adverse molecular markers [other than CEBPA (+)], occurred in 13.2% of primary AML patients and was closely associated with older age, intermediate-risk cytogenetics, NPM1 mutation and ASXL1 mutation (40). In our study, ASXL1 and TET2 were present at higher levels in the AML with BMF group, which predicted poor prognosis. Mutations of the FMS-like tyrosine kinase 3 (FLT3) gene appear in approximately 30% of AML cases, which is the only druggable molecular abnormality today that help patients achieve longer and more durable remissions. FLT3 with internal tandem duplication (FLT3-ITD) is the most common type of FLT3 mutation in AML, which presents with a high leukemic burden and a poor prognosis. While FLT3 mutation in the tyrosine kinase domain (FLT3-TKD) has a lower incidence, and the prognostic value of FLT3-TKD is uncertain (41). Our study showed the mutation frequencies of FLT3-ITD and FLT3-TKD had no statistically significant differences between the two groups. CCAAT/enhancer-binding protein-alpha (CEBPA), a transcription factor, regulates the proliferation and differentiation of myeloid progenitors. One study found that patients with the CEBPA mutation had favorable outcomes in the absence of any other prognostic factors indicating a poor outcome. Systematic analysis of CEBPA mutations, as well as changes in hematopoietic master genes, may be helpful in assessing the prognosis of AML, especially for patients in the “intermediate” prognosis subgroup (42). The mutation frequency of CEBPA in our study was significantly lower in the AML with BMF group. Therefore, we hypothesized that AML patients with BMF had a poor prognosis. However, accurate assessment of prognosis and risk stratification of AML patients requires the consideration of coexisting mutations. A study performed by Papaemmanuil et al. showed that there were interactions among genes, so the commutation-identified groups determined a favorable or adverse prognosis (43). Therefore, the effect of the coexistence of multiple gene mutations should be fully considered when assessing the risk stratification of AML, and the impact of complex genomic changes on clinical prognosis should not be ignored.

The CR/CRi rate and OS of AML patients with different induction therapies were statistically analyzed. Our study showed that there was no significant difference in CR/CRi rate and OS among the IA, DA and CAG groups, which excludes their influence on our study.

Multivariate analysis showed that BMF had independent prognostic significance. AML patients without BMF had a higher CR/CRi rate, and the time of hematopoietic recovery in patients achieving CR/CRi was longer in BMF group. The degree of BMF, prognostic level and blasts in peripheral blood were independent risk factors for CR/CRi in newly diagnosed AML patients. Therefore, early screening of AML patients with BMF, genetic and chromosomal examinations are of great significance for the development of individualized treatment regimens, improvement of clinical efficacy and outcome.

The correlation between BMF and the prognosis in newly diagnosed AML patients is controversial. Manoharan et al. thought that increased marrow reticulin did not change the overall prognosis of acute leukemia and that effective antileukemia therapy could reduce bone marrow reticulin (17). However, another study reported that moderate to marked marrow reticulin in patients with acute leukemia predicted a poor outcome, which was attributed to the persistence of marrow reticulin and possible interference with the normal hematopoietic reconstruction of the bone marrow after chemotherapy (44). Wu et al. confirmed that BMF was an independent risk factor for the survival of AML patients (18). Our research showed that AML patients with BMF had a lower OS rate, especially AML patients with BMF ≥ 2, indicating that BMF was an independent prognostic factor affecting the OS of AML patients. These results suggest that AML and BMF jointly affect the prognosis of AML patients with BMF.

In our study, Cox multivariate analysis showed that age had independent prognostic significance for the OS of primary AML patients. Therefore, AML with or without BMF was divided into two groups aged less than 60 years and greater than or equal to 60 years. AML with the BMF group had a lower OS rate, regardless of age < 60 or ≥ 60 years old. However, in AML patients younger than 60 years old, the higher the degree of BMF was, the shorter the median survival time and the lower the OS rate. In the age ≥ 60 group, the median survival time in the BMF-1 and the BMF-2/3 groups was shorter.

In addition, survival analysis by different risk stratifications was performed. For primary AML patients with low, intermediate and high risk, there was always a lower OS rate in patients with BMF. The median survival of AML patients decreased with an increasing degree of fibrosis in different risk stratifications, which suggested that the conventional chemotherapy regimen could not effectively improve the OS of AML patients with BMF. Therefore, we need to optimize the chemotherapy regimen to improve the survival time of AML patients with BMF.

However, bone marrow biopsy was not a routine examination for newly diagnosed AML. In addition, bone marrow biopsy was performed only when the presence of BMF was considered at the initial diagnosis of AML in the past. Therefore, the data collected included more patients with BMF than those without BMF. In addition, because our study was a retrospective and monocentric study, the conclusions may be biased. In the future, we will conduct bone marrow biopsy for each newly diagnosed patient to further expand the sample size and further verify our conclusions.

## Conclusion

In conclusion, our study showed that AML patients with BMF have a poor prognosis. We found that BMF and age were independent prognostic factors affecting the OS of AML patients. Hence, bone marrow biopsy should be a routine examination during the diagnosis of AML. More studies are needed to confirm that BMF could be used as an important predictor of risk stratification in AML patients. Further research on the pathophysiological mechanism of bone marrow is of great significance for determining the prognostic risk stratification of AML patients with BMF, developing appropriate chemotherapy regimens and improving the clinical efficacy of treatment.

## Data availability statement

The original contributions presented in the study are included in the article/supplementary material, further inquiries can be directed to the corresponding author/s.

## Ethics statement

Written informed consent was obtained from the minor(s)’ legal guardian/next of kin for the publication of any potentially identifiable images or data included in this article.

## Author contributions

XZ and ZJ conceived and designed the experiments. JY, XZ, and FW collected clinical samples and data. XZ wrote the manuscript. All authors contributed to the article and approved the submitted version.

## Funding

This work was supported by the Joint Construction Project of the Henan Province Medical Science and Technology Research Plan (Grant NO. LHGJ 2018020048).

## Conflict of interest

The authors declare that the research was conducted in the absence of any commercial or financial relationships that could be construed as a potential conflict of interest.

## Publisher’s note

All claims expressed in this article are solely those of the authors and do not necessarily represent those of their affiliated organizations, or those of the publisher, the editors and the reviewers. Any product that may be evaluated in this article, or claim that may be made by its manufacturer, is not guaranteed or endorsed by the publisher.
